# Transforming guided internet interventions into simplified and self-guided digital tools – Experiences from three recent projects

**DOI:** 10.1016/j.invent.2023.100693

**Published:** 2023-11-23

**Authors:** Martin Kraepelien, Amira Hentati, Dorian Kern, Christopher Sundström, Susanna Jernelöv, Nils Lindefors

**Affiliations:** a Centre for Psychiatry Research, Department of Clinical Neuroscience, Karolinska Institutet, Stockholm Health Care Services, Region Stockholm, Sweden; b Department of Psychology, Uppsala University, Uppsala, Sweden; c Division of Psychology, Department of Clinical Neuroscience, Karolinska Institutet, Stockholm, Sweden

**Keywords:** ISRII, Pittsburgh, Self-guided, Digital self-care, Digital tools, Stepped care

## Abstract

**Introduction:**

Therapist-guided internet interventions are often more efficacious than unguided ones. However, the guidance itself requires clinician time, and some research suggests that self-guided interventions could potentially be equally effective. The concept of digital psychological self-care, self-guided internet interventions based on the use of digital tools and provided within a structured clinical process, is presented.

**Methods:**

Three new self-care interventions, a sleep diary-based intervention for insomnia, an alcohol diary-based intervention for problematic alcohol use and an intervention with exposure and mindfulness tools for atopic dermatitis (eczema), were developed. Newly developed digital self-care interventions were compared to the earlier therapist-guided interventions they were based on, using published results from three feasibility trials (n's = 30, 36 and 21) and three randomized trials (n's = 148, 166 and 102). The comparison included type of content, duration, length of written material and within-group effect-sizes.

**Results:**

In comparison to the guided interventions, clinician time was greatly reduced and the new interventions involved much less reading for participants. The digital self-care tools also showed within-group effect sizes and response rates on par with the more comprehensive guided internet interventions.

**Discussion:**

Preliminary results suggest that some guided internet interventions can be transformed into self-guided digital tools. These three examples show that digital psychological self-care, if provided with telephone interviews before and after the intervention, can be viable alternatives to more comprehensive guided internet interventions. Although these examples are promising, further studies, including randomized experiments, are needed to compare treatment efficacies, and to identify which groups of patients may need more comprehensive guided internet interventions.

## Highlights

• Three self-care versions of guided internet interventions are presented.
• Self-care had shorter duration, fewer words and a narrower focus compared to guided.
• Automated messages and clinical interviews were used to promote adherence.
• Self-care used less clinician time than guided and had promising preliminary effects.
• Randomized studies are needed to compare treatment efficacy.

## 1 Introduction

Therapist-guided internet interventions based on Cognitive Behavioral Therapy (CBT) are usually more efficacious than self-guided interventions. However, therapist guidance consumes clinician time which can hinder implementation (Andersson et al., 2019; Hedman et al., 2012; Karyotaki et al., 2018). Some studies on depression and anxiety, however, suggest no consistent differences in effects between self-guided and therapist-guided interventions when the self-guided intervention is of high quality and delivered within a structured care process (Titov et al., 2015). Aspects considered important for the success of self-guided interventions are clinical interviews conducted before and after the intervention, clinical monitoring during the intervention, well-designed interventions and standardized automated reminders and messages (Titov et al., 2013, Titov et al., 2015).

In an ongoing development project at Karolinska Institutet and Region Stockholm, Sweden, new digital self-guided interventions are being developed, often as briefer versions of previous, more comprehensive therapist-guided interventions. This concept has come to be called *digital psychological self-care*. Important aspects of this concept are that the self-guided interventions:

• are provided together with brief clinical interviews before and after the intervention.
• have a content that is brief and concise.
• include one or two main digital tools that the user is encouraged to focus on.
• have an easy-to-use graphical user interface (GUI).
• are complemented with automated reminders and messages.

Previous results from this development project include a large randomized controlled trial showing that an optimized GUI with automated features and stepwise presentation of content facilitate behavioral engagement to a greater degree than a previous, more basic user interface (Hentati et al., 2021).

The aim of this report is to briefly describe three digital self-care interventions within the current development project, using data from three newly published feasibility studies. The aim is also to compare the self-guided interventions to three corresponding guided and more comprehensive interventions with published randomized trials, using these cases to describe the concept of digital psychological self-care interventions.

## 2 Methods

First, the three self-care interventions will be presented. The new interventions will then be compared to earlier guided interventions in terms of content, duration and length of written material. These earlier interventions were all internet-based CBT provided with guidance and consisting of self-help texts and exercises roughly corresponding to a self-help book in length. For more information on these interventions and the participants included in the comparisons, please see Table 2, Table 3, Table 4, and the referenced earlier studies below. We also present within-group effects from the three recently completed small feasibility studies.

A self-care intervention for insomnia (Jernelöv et al., 2023) will be compared to a guided CBT-intervention, previously compared to an active control condition in a randomized trial (Kaldo et al., 2015). In that study, the guided intervention was significantly more effective than the control treatment in reducing ISI (Cohen's *d* = 0.85). A self-care intervention for problematic alcohol use (Kraepelien et al., 2023) will be compared to a guided CBT-intervention previously compared to an active control condition, and a wait-list, in a randomized trial (Sundström et al., 2020). The guided intervention showed superiority against the wait-list condition regarding effects on alcohol consumption (Cohen's *d* = 0.78), as did the active control intervention (Cohen's *d* = 0.48), but the two active interventions did not differ significantly in this study. A self-care intervention for atopic dermatitis (Kern et al., 2022, Kern et al., 2023a) will be compared to a guided CBT-based intervention previously compared to a control condition consisting of standard care instructions, in a randomized trial (Hedman-Lagerlöf et al., 2021). The guided intervention was significantly more effective than the control condition in reducing symptoms of atopic dermatitis (Cohen's *d* = 0.75). When data on intervention word length was not available in the published studies, the intervention texts were pasted into a word processor and words were counted. Within-group effects were calculated with 95 % confidence intervals from treatment initiation to the 3-month follow-up, except for one case (the therapist-guided intervention for insomnia) where the 6-month follow-up was used due to a 3-month follow-up not being included in the study. For the comparison of insomnia, the Insomnia Severity Index (ISI; Bastien et al., 2001) was used to calculate within-group effects, and a responder was defined as having a change-score of 8 points or more on the ISI. For the comparison of problematic alcohol use, number of standard drinks consumed during each of the preceding seven days (Sobell et al., 1979) was used as outcome. There was no consistent definition of response in the two studies of the alcohol comparison, thus number of responders were not reported. For the comparison of atopic dermatitis, the Patient Oriented Eczema Measure (POEM; Schram et al., 2012). score was used, and a responder was defined as having a change-score of 4 points or more on the POEM. Participants in all six studies were primarily recruited in the general public with the help of online advertisements. No participants were compensated monetarily in any of the studies.

**Fig. 1:**
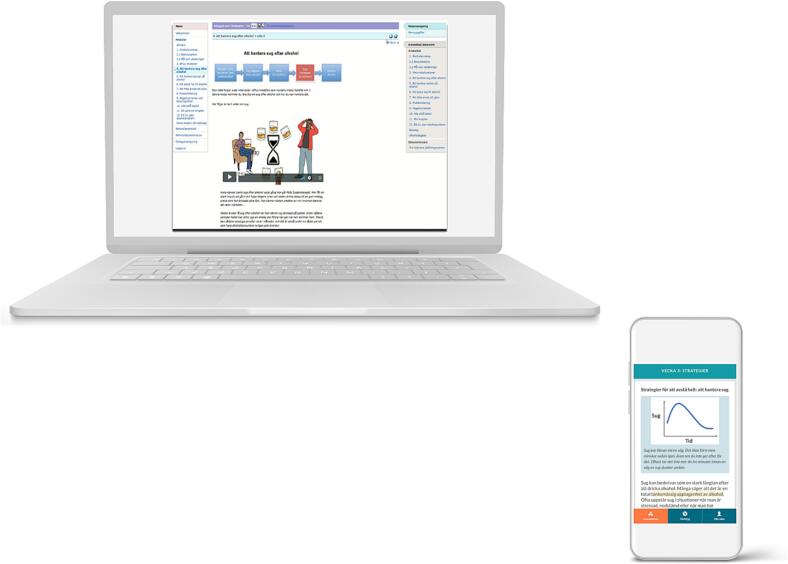
A comparison between the old, basic GUI (top left) and the new, optimized “mobile first” GUI (bottom right).

In all three examples, the following was part of the clinical routine for the self-care interventions: telephone interviews before and after the program to motivate the participant, automated messages, automated unlocking of new modules, and a new optimized mobile-friendly GUI. See Fig. 1 for examples of how the optimized GUI differ from the previous, older version of the GUI, and Table 1 for conceptual similarities and differences between the therapist-guided interventions and the self-care interventions. The GUI was developed in collaboration with experts on user experience (Hentati et al., 2021).

**Table 1:** Conceptual similarities and differences between the therapist-guided interventions and the new self-guided digital tools.

	Therapist-guided intervention	Self-guided digital tool
Telephone interviews	Before and after intervention	Before and after intervention
Means of support	Personalized therapist messages	Generalized automated messages
Unlocking new modules	Opened by therapist	Automated if/when: 1 week had passed + participant finished module
Type of homework	New work sheet every week	Same tool throughout intervention
Responsive design	Desktop first	Mobile first

All three self-care interventions focused on one or two tools based on core components of interventions conventionally used in CBT for the current condition. What core components to base the tools on and what to leave out was decided by the authors of the studies based on clinical experience and the literature on CBT for that particular problem. Components of CBT left out from the main tools were still mentioned as optional ways to handle the problem within the interventions. For more information on the components of the self-care interventions, please see the published studies on insomnia (Jernelöv et al., 2023), problematic alcohol use (Kraepelien et al., 2023) and atopic dermatitis (Kern et al., 2022, Kern et al., 2023a).

## 3 Results

### 3.1 Digital self-care for insomnia

The sleep diary tool was the main component of the insomnia intervention. In line with sleep restriction therapy, it automatically guided the participant to set a sleep window, register sleep, and review and adjust the sleep window (Jernelöv et al., 2023). A daily automated text message in the morning reminded the participant to register the sleep of the previous night. Other components that were included in the full CBT-package of the therapist guided insomnia intervention, for example relaxation, visualization and managing thoughts (Kaldo et al., 2015), were only briefly mentioned and did not include features such as work-sheets which were part of the original treatment. Table 2 presents a comparison between the two insomnia interventions. The within-group effect size of 1.81 at the follow-up was within the 95 % confidence interval of the corresponding effect size from the therapist-guided intervention.

**Table 2:** Comparison between the therapist-guided intervention and the self-guided digital tool for insomnia.

	Therapist-guided intervention (Kaldo et al., 2015)	Self-guided digital tool (Jernelöv et al., 2023)
Number of study participants	*n* = 73 + 75	*n* = 30
Mean age in study	48	46
Gender proportion in study	78 % female	63 % female
Education level in study	22 % secondary school 74 % university 4 % other	13 % secondary school 87 % university 0 % other
Insomnia severity index mean at intervention start	Intervention group = 16.8 Control group = 16.5	16.5
Number of words	≈80,000	10,500
Duration	8 weeks	4 weeks
Content	Full CBT for insomnia-package including new content and homework exercises every week, with a broad CBT-focus	Focused on sleep restriction-therapy
Therapist guidance	M = 115 min/participant	0 min by design
Within-group effect on ISI at 3-month follow-up	*d* = 1.71 (95 % CI = 1.33–2.08) (6-month follow-up)	*g* = 1.81 (95 % CI = 1.20–2.42)
Responders at follow-up	45 %	41 %

### 3.2 Digital self-care for problematic alcohol use

The alcohol diary tool was the main component in the alcohol intervention. It automatically guided the participant to set consumption goals and register consumption daily. As in the sleep diary tool, participants were reminded to register consumption by daily automated text messages. Some components from the earlier more comprehensive alcohol intervention (Sundström et al., 2020), such as analyzing risk-situations, finding other activities and using strategies for handling cravings, were also present as resources within the self-care intervention, though the central task was registering consumption (Kraepelien et al., 2023). The earlier comprehensive intervention also included CBT-components such as problem solving and cognitive restructuring that was not used in the self-care intervention. Moreover, the self-care intervention concentrated all new concepts to the first 4 weeks while week 5 to 8 consisted of setting a new goal, keep registering consumption and practicing the content learned during the first weeks. Table 3 presents a comparison between the two alcohol interventions. The within-group effect size of 0.70 at the follow-up was within the 95 % confidence interval of the corresponding effect size from the therapist-guided intervention.

**Table 3:** Comparison between the therapist-guided intervention and the self-guided digital tool for problematic alcohol use.

	Therapist-guided intervention (Sundström et al., 2020)	Self-guided digital tool (Kraepelien et al., 2023)
Number of study participants	*n* = 72 + 71 + 23	*n* = 36
Mean age in study	53	50
Gender proportion in study	51 % female	58 % female
Education level in study	36 % secondary school 57 % university 7 % other	39 % secondary school 61 % university 0 % other
Initial alcohol use disorder severity in study	0 % subthreshold 8 % mild 17 % moderate 75 % severe	8 % subthreshold 14 % mild 39 % moderate 39 % severe
Number of words	≈40,000	21,500
Duration	12 weeks	8 weeks
Content	New content and homework exercises every week with a broad CBT-focus	Focused on goal-setting and registration of drinks with new content only the first four weeks
Therapist guidance	m = 162 min/participant	0 min by design
Within-group effect on drinks per week at 3-month follow-up	*d* = 0.46 (95 % CI = 0.13–0.80)	*g* = 0.70 (95 % CI = 0.19–1.21)

### 3.3 Digital self-care for atopic dermatitis

Both the old (Hedman-Lagerlöf et al., 2021) and the new (Kern et al., 2022, Kern et al., 2023a) interventions for atopic dermatitis (eczema) had exposure (with response prevention) and mindfulness as main components. Exercises included neutrally observing bodily sensations without judging them, to provoke symptoms, approach challenging social situations, and to challenge unhelpful avoidance behaviors (Kern et al., 2023a). The difference in participant work load between the two interventions was considerable, with the old, more comprehensive intervention using over 40 work sheets. These work sheets covered a number of additional interventions, including diaries of feelings of itch and scratching behavior, functional behavioral analysis, and cognitive exercises. By contrast, the new intervention was centered around a tool with a library of mindfulness exercises and a tool that helped plan and register exposure exercises, often with the response prevention of not scratching. Unlike in the self-care versions for insomnia and alcohol, daily text message reminders were not used for this self-care intervention. Table 4 presents a comparison between the two atopic dermatitis interventions. The within-group effect size of 0.84 at the follow-up was within the 95 % confidence interval of the corresponding effect size from the therapist-guided intervention.

**Table 4:** Comparison between the therapist-guided intervention and the self-guided digital tool for atopic dermatitis (eczema). An earlier version of this table is published in the research letter by Kern and colleagues (Kern et al., 2022).

	Therapist-guided intervention (Hedman-Lagerlöf et al., 2021)	Self-guided digital tool (Kern et al., 2022, Kern et al., 2023a)
Number of study participants	*n* = 51 + 51	*n* = 21
Mean age in study	37	43
Gender proportion in study	81 % female	96 % female
Education level in study	23 % secondary school 69 % university 9 % other	28 % secondary school 61 % university 11 % other
Initial eczema severity in study	1 % mild 38 % moderate 44 % severe 17 % very severe	14 % mild 24 % moderate 43 % severe 19 % very severe
Number of words	111,000	16,500
Duration	12 weeks	8 weeks
Content	Extensive psychoeducation and new homework exercise work sheets every week	Brief psychoeducation and focused on the same mindfulness and exposure exercises during all weeks
Therapist guidance	m = 40 min/participant	0 min by design
Within-group effect on POEM at 3-month follow-up	*d* = 0.89 (95 % CI = 0.50–1.28)	*d* = 0.84 (95 % CI = 0.38–1.37)
Responders at follow-up	65 %	69 %

## 4 Discussion

These three cases of new self-care versions of older, more comprehensive therapist-guided interventions showed that the concept of digital psychological self-care (brief self-guided internet interventions provided within as structured care process) is promising. The self-care interventions used a new optimized GUI, less amount of text, had a shorter duration, and no therapist-guidance compared to the full interventions which they were based on. Text content was reduced to as little as 15 % of the earlier guided internet interventions. Time spent by a clinician was also greatly reduced compared to earlier interventions, although time was still used for brief clinical interviews before and after the intervention, hypothesized to boost compliance to core components. The self-care intervention for problematic alcohol use is the only one of the compared interventions where there is data on the actual average length of these clinical interviews that was on average 34 min for the initiating interview and 22 min for the post-interview (Kraepelien et al., 2023). These interviews were probably shorter in the case of atopic dermatitis where no psychiatric diagnostic interview where performed. Although the studies did not include a common measure of engagement, engagement with core components was indeed high in general in the self-care interventions: In the insomnia intervention, 90 % of participants registered half of the nights or more in the sleep diary tool (Jernelöv et al., 2023). In the alcohol intervention, participants completed on average 76 % of daily consumption registrations signaling a high degree of daily use of the main tool (Kraepelien et al., 2023). In the intervention for atopic dermatitis, 65 % of participants returned at least 5 out of 8 homework assignments (Kern et al., 2022). The promising levels of engagement observed could be partly explained by the content being digestible and being presented in a visually appealing design, factors that users have stated as important in a survey of users of self-guided interventions (Gan et al., 2023). Another explanation may be that the brief clinical interviews before and after the intervention was efficient in increasing engagement through accountability to the clinician (Mohr et al., 2011) in the same way as therapist-guidance may work in guided interventions.

The preliminary within-group effects were promising and similar to the therapist-guided interventions, as were response rates for the insomnia and atopic dermatitis comparisons. However, these were not direct comparisons, and samples from the studies on self-care interventions were small and confidence intervals large, which call for caution of drawing any conclusions. Samples were rather similar demographically, and in initial severity, in each comparison, with the exception of the alcohol comparison where the participants in the study of the guided intervention had more severe levels of alcohol use disorder initially. These differences further limit the conclusions that can be drawn on the possible similarity of within-group effects between these two studies. See the feasibility study of digital self-care for problematic alcohol consumption for an extended discussion on the differences between these two samples (Kraepelien et al., 2023). There is no information on ethnicity in neither of the three comparisons due to this not being the tradition to assess in Swedish studies. However, we can say with relative certainty that since good knowledge of Swedish was an inclusion criterion in all studies, the part of the Swedish migrant population that does not have sufficient knowledge of Swedish was to some extent prevented from participating in the studies. However, the less extensive text length of the self-care interventions may facilitate participation in the interventions for people with lower language skills in Swedish as well as facilitate future translations and cultural adaptations of the material.

The post-intervention interviews in the three feasibility studies of self-care interventions included opportunities for the participants to comment on the content and design of the interventions. These comments from participants are being used in revising the interventions for the studies and implementation projects that may be the next steps for these interventions. Further studies, including randomized experiments, are needed to compare treatment efficacy and to identify which groups of patients may need the more comprehensive and therapist-guided internet interventions. For example, there is a non-inferiority trial planned, comparing the two interventions for atopic dermatitis in a randomized experiment (Kern et al., 2023b). If compliance to core components of interventions, and clinical outcomes, are shown to be similar to those in therapist-guided internet interventions, these self-care interventions can be an easily administered first step in a stepped care system for health care services in the treatment of many different conditions.

## Funding

This work was supported by the 10.13039/501100005348Swedish Ministry of Health and Social Affairs (grant number S2018/03855/FS) and 10.13039/501100006636FORTE (grant number 2020-01160).

## Declaration of competing interest

The authors declare that they have no known competing financial interests or personal relationships that could have appeared to influence the work reported in this paper.
